# Hearing problems in humans and mouse models with rare copy number variants associated with schizophrenia: a scoping review protocol

**DOI:** 10.12688/wellcomeopenres.23013.1

**Published:** 2024-09-24

**Authors:** Stephen Murtough, Daniele Panconesi, Chen Lu, Rosemary Abidoph, Marius Cotic, Daisy Mills, Alvin Richards-Belle, Maria Richards-Brown, Noushin Saadullah Khani, Lauren Varney, Jennifer F Linden, Elvira Bramon

**Affiliations:** 1 Mental Health Neuroscience Department, Division of Psychiatry, University College London, London, W1T 7NF, UK; 2 Central and North West London NHS Foundation Trust, London, NW1 3AX, UK; 3 Ear Institute, University College London, London, WC1X 8EE, UK; 4 Department of Genetics and Genomic Medicine, Great Ormond Street Institute of Child Health, University College London, London, WC1N 1EH, UK; 5 Epidemiology and Applied Clinical Research Department, Division of Psychiatry, University College London, London, W1T 7NF, UK; 6 Department of Neuroscience, Physiology & Pharmacology, University College London, London, WC1E 6BT, UK; 7 Camden and Islington NHS Foundation Trust, London, NW1 0PE, UK

**Keywords:** Peripheral hearing loss, central hearing loss, hearing problems, hearing impairment, copy number variants, schizophrenia, psychosis, auditory evoked potentials, mouse models

## Abstract

**Background:**

Hearing loss is a risk factor for developing auditory hallucinations and other psychosis symptoms. To date, very little research has investigated hearing loss in individuals with a high genetic risk of developing schizophrenia and other types of psychosis. 13 copy number variant (CNV) loci are robustly associated with an increased risk of schizophrenia. Of these, microdeletions at 22q11.2 often lead to some hearing loss, and mouse models of this CNV display impaired auditory functioning. We hypothesise that hearing impairment is an understudied mechanism that contributes to the formation of psychosis symptoms in individuals at high genetic risk. This scoping review will explore whether the 13 schizophrenia-associated CNVs are related to hearing problems, including peripheral hearing loss and other auditory problems, in humans and related mouse models.

**Methods:**

Our scoping review will follow guidelines provided by the Joanna Briggs Institute and the Preferred Reporting Items for Systematic reviews and Meta-Analyses extension for Scoping Reviews. A systematic search will be completed using PubMed (MEDLINE), Embase, PsychINFO, and Cochrane Library databases, as well as other sources to identify relevant grey literature. Search terms will include all commonly used synonyms for hearing loss and problems with auditory perception, and both human and mouse studies that describe relevant CNVs will be included. Search lists will be screened by two authors independently, according to eligibility criteria, and data will be extracted and summarised using a narrative approach, and meta-analysis methods will be used if possible.

**Conclusions:**

To our knowledge, this will be the first scoping review to explore auditory functioning across all CNVs that confer high schizophrenia risk. Looking ahead, if hearing problems are a clinical feature in these groups (including humans and related mouse models), they may serve as useful genetic models for future mechanistic studies.

## Plain language summary

Hearing loss can increase the likelihood of hearing voices and sounds that aren’t real, which we call auditory hallucinations. These hallucinations are commonly experienced by people with schizophrenia, which is a serious mental health condition. Some people have a higher chance of developing schizophrenia, due to having specific genetic variations, and one of these genetic differences can also cause hearing loss. We want to investigate whether people with 12 other genetic variations also have hearing loss and other hearing problems, as this may be a key reason why they experience auditory hallucinations. We have developed a strategy to scan the medical literature for studies that assess people with these variations, as well as mice with comparable changes. If hearing problems are observed in these people and/or mice, we plan to carry out future studies to better understand why these genetic variations can lead to distressing auditory hallucinations in some people.

## Introduction

Schizophrenia is commonly associated with psychosis symptoms, including auditory hallucinations ^1^. Risk of developing schizophrenia is linked to an individual’s genetics and environmental exposures ^1^, although an overlooked risk factor is hearing loss ^2– 5^. A meta-analysis found that hearing impairment was linked to an increased risk of psychosis symptoms, including hallucinations, delusions, and delirium ^2^. And a recent study found that 16.2% of hearing-impaired individuals experienced auditory hallucinations, which increased to 24% in those with severe impairment ^3^. The mechanisms that drive these processes remain largely unexplored, and an opportunity could be to study individuals who are at high genetic risk.

 Considering this, genetic risk for schizophrenia is determined by common risk loci ^6^, rare coding variants ^7^, and rare copy number variants (CNVs) ^8^. In more detail, 13 CNVs significantly increase risk of schizophrenia (shown in Table 1), and 8 have genome-wide significance (with odds ratios between 3.8 and 67) ^8^. Prevalence of the 13 CNVs in the general population is low ^8^, but an understanding of these may inform about the biological mechanisms of psychosis symptom formation more generally ^9^.

**Table 1.** Summary of 13 CNVs that significantly increase risk of schizophrenia (adapted from Marshall et al., 2017 ^8^). In the first column, genes that place in specific loci are shown in brackets, and where two CNVs are present at a locus, “Proximal” and “Distal” terms are used. The top 8 listed CNVs have genome-wide significance ^8^.

Genomic Locus (Gene)	Type of CNV	Odds Ratio
22q11.2	Loss	67.7
16p11.2, Proximal	Gain	9.4
2p16.3 ( *NRXN1*)	Loss	14.4
15q13.3	Loss	15.6
1q21.1	Loss + Gain	3.8
3q29	Loss	Not available
16p11.2, Distal	Loss	20.6
7q11.23	Gain	16.1
Xq28, Distal	Gain	8.9
15q11.2	Loss	1.8
9p24.3 ( *DMRT1*)	Loss + Gain	12.4
8q22.2 ( *VPS13B*)	Loss	14.5
7p36.3 ( *VIPR2*, *WDR60*)	Loss + Gain	3.5

 Microdeletions at 22q11.2, which are commonly known as 22q11.2 deletion syndrome (DS; also known as DiGeorge Syndrome and Velocardiofacial Syndrome), are one of the best characterised schizophrenia-associated CNVs. 22q11.2 DS is linked to varied health conditions, including multiple organ dysfunction, intellectual disability, and immunological abnormalities ^10^, and lifetime risk of schizophrenia in these individuals is ~25%, which makes it one of the strongest known genetic risk factors ^11^. Furthermore, hearing impairment (mainly conductive hearing loss) is a common feature of 22q11.2 DS ^12– 14^, and mouse models of 22q11.2 DS also display hearing abnormalities ^15– 17^.

 While hearing loss in 22q11.2 DS is a recognised clinical feature, hearing function in individuals with other schizophrenia-associated CNVs remains largely unexplored, and no synthesis of the literature is available on this topic. We hypothesise that (some of) the 13 CNVs are linked to hearing problems (that may be peripheral or central in origin), which may contribute to psychosis symptom formation. This scoping review aims to explore this hypothesis. We will systematically review the literature, including studies on both humans and related mouse models, and we will focus on peripheral hearing loss (defined as conductive, sensorineural, and mixed), while also considering other hearing problems that may be central in origin, such as hyperacusis. We hope that our synthesis will inform future genetics and mechanistic studies.

## Protocol

This protocol has been designed using method guidelines provided by the Joanna Briggs Institute ^18^ and the Preferred Reporting Items for Systematic reviews and Meta-Analyses extension for Scoping Reviews (PRISMA-ScR) ^19^. Full details (that allow for study reproducibility) are shown below. In addition, a PRISMA for Protocols (PRISMA-P) checklist ^20^ has been completed and relevant sections for scoping reviews have been addressed (Extended data).

### Research questions

1) Are CNVs that increase risk of schizophrenia associated with hearing problems in human and mouse studies?
2) If hearing problems are observed, what is their nature?*

*This will include peripheral hearing loss (defined as conductive, sensorineural, and mixed) and any other auditory problems that may be central in origin.

### Eligibility criteria

***Participants***
1) Human participants with a confirmed CNV at a genomic locus that is robustly associated with an increased risk of schizophrenia (which includes 13 CNV loci, shown in Table 1) ^8^. Participants can be any age, gender, ethnicity, and from any geographical region.
2) Mouse models of human CNVs that confer increased risk of schizophrenia (if they exist) ^8^. Mouse models can be any strain or genetic background, and this information will be recorded, as some genetic backgrounds can exhibit age-related hearing loss.
***Concept***
1) The main concept of this review is the presence or absence of any type of hearing problem in participants (humans and mouse models) with schizophrenia-associated CNVs.
2) This review will consider all types of peripheral hearing loss (sensorineural, conductive, and mixed), as well as other auditory problems that may be central in origin (such as hyperacusis). Any presence of ear malformations, ear damage, and/or ear infections will also be considered, as these may lead to hearing impairment.
2) In mouse studies (and human studies, if available), auditory evoked potentials may be considered as a proxy for determining hearing function. Comparisons to human studies will be made cautiously, and the origin of hearing problems (peripheral or central) will be considered.
***Context***
The context of this review is intentionally broad and is limited only to studies describing humans and mouse models with schizophrenia-associated CNVs. No restrictions will be applied to publication period, geographical region, study design, or language.
***Types of sources***
All study designs will be considered in this scoping review, but the search will be performed using English language. We expect studies will be mainly descriptive reports of family pedigrees, including case reports and series, as well as experimental studies of related mouse models. Grey literature will also be considered, including articles uploaded to preprint servers.

### Search strategy

Each schizophrenia-associated CNV will be searched for individually to minimise human error when extracting data. The search strategy will involve four stages:

1) An exploratory search for each schizophrenia-associated CNV (e.g., “22q11.2”) will be performed using PubMed. This will identify alternatively used names, such as DiGeorge Syndrome for 22q11.2 DS, which will inform the final search string.
2) A systematic search for eligible studies will be completed using PubMed (MEDLINE), Embase, PsychINFO, and Cochrane Library databases. Each CNV will be searched for individually, and the search string will include the name of the CNV locus (and any alternatively used names); all commonly used synonyms for hearing loss, including “hearing impairment” and “deaf”; as well as terms describing other types of auditory problem that may be central in origin, including “hyperacusis” and “auditory processing disorder”. An example search string for 22q11.2 is shown in Table 2.
3) Searches for relevant grey literature will be performed for each CNV using Google Scholar, preprint servers (including medRxiv), and search engines.
4) For studies included for data extraction (which is described below), reference lists will be screened to ensure all relevant articles are captured, and a forward citation search will be performed using Google Scholar.

**Table 2.** Example search string using PubMed. Search performed on 21 ^st^ August 2024. Multiple word phrases are included in quotation marks, and word truncation is denoted by*.

Example search string using PubMed	Search results (No.)
(22q11.2 OR “DiGeorge Syndrome” OR “Velocardiofacial Syndrome”) AND (hearing * OR “hearing loss” OR “hearing impairment” OR auditor * OR “auditory impairment” OR “auditory evoked potential *” OR “auditory brainstem response *” OR audio OR “auditory hypersensitivity” OR “auditory processing disorder” OR hyperacusis OR deaf * OR sensorineural OR “conductive hearing” OR “mixed hearing” OR ear OR ears OR “ear infection *” OR auricular OR otitis OR “otitis media” OR otolog * OR otorhinolaryngolog * OR otolaryngol * OR cochlear OR vestibulocochlear OR “cranial nerve VII *” OR “cranial nerve eight” OR “eighth cranial nerve” OR “facial nerve” OR “cranial nerve seven” OR “seventh cranial nerve”)	418

For the above search strategy, no restrictive publication period or language requirements will be applied. Any non-English articles will be translated using DeepL.

### Study selection

Studies captured in the search will be exported and uploaded onto the Rayyan platform ( https://www.rayyan.ai/). Here, studies will be independently screened by two reviewers (SM and DP) according to eligibility criteria. Initial screening by the reviewers will remove duplicates and any obviously irrelevant studies (based on title and abstract), and the remaining studies will be screened by assessing the full text. Relevant conference abstracts will also be screened, and authors will be contacted for more information if required. Any disagreements over whether to include a study will be resolved by consulting a third and fourth reviewer (EB and JL).

### Data extraction

Data will be extracted from studies into an Excel spreadsheet, and these will include:

***General study details***
Study title, authors and affiliations, journal information, publication year, and study design.
***Specific study details***
CNVs that are studied, species, sample characteristics (incl. age, sex, ethnicity), sample size, mouse strain and genetic background (if applicable), type of hearing problem, origin of hearing problem (peripheral or central, if identifiable), measures of hearing function, and any additional notes of interest.

We will attempt to fill any missing information by contacting study authors.

### Data analysis

Data from studies will be summarised using a narrative approach. Any quantitative measures of hearing impairment will be presented and described, and if sufficient studies with comparable quantitative outcomes are identified, we will conduct a meta-analysis. We anticipate findings to be summarised in text and using tables. Both research questions will be addressed using this approach. If sufficient study numbers are identified, results will be presented separately for human and mouse studies.

## Discussion

Hearing loss is a risk factor for developing auditory hallucinations and other psychosis symptoms ^2– 5^. We hypothesise that high-risk genomic variants for schizophrenia could provide mechanistic insights, although it is largely unknown whether these lead to hearing problems. This scoping review will provide a first exploration and synthesis of the literature on this topic, by focusing on 13 schizophrenia-associated CNVs. 

 A strength of this review will be its broad assessment of hearing loss and function (including terms for both peripheral and central abnormalities), and its inclusion of both human and mouse studies. We hope this will improve study numbers, particularly as the prevalence of schizophrenia-associated CNVs is low in the general population. A potential limitation might be that hearing function is not reported in phenotypic studies, or that hearing issues are not clinically obvious during assessments. To partially navigate this, we have included all known schizophrenia-associated CNVs, to capture all relevant studies in the literature, and we will contact study authors when we have questions about any findings.

 We hope this scoping review will help to determine whether schizophrenia-associated CNVs are related to hearing problems, which may provide a springboard for future genetics and mechanistic studies.

## List of abbreviations

22q11.2 DS = 22q11.2 deletion syndrome

CNV(s) = Copy Number Variant(s)

PRISMA-P = Preferred Reporting Items for Systematic reviews and Meta-Analyses extension for Protocols

PRISMA-ScR = Preferred Reporting Items for Systematic reviews and Meta-Analyses extension for Scoping Reviews

## Ethics

This scoping review (and its protocol) does not require ethical approval.

## Data Availability

No data are associated with this article. **Open Science Framework:** Hearing problems in humans and mouse models with rare copy number variants associated with schizophrenia: a scoping review protocol. https://doi.org/10.17605/OSF.IO/S9NQG ^21^. **This project contains the following extended data:** Appendix 1: A completed PRISMA-P checklist for the scoping review protocol. Data License: CC-By Attribution 4.0 International
