# Supplementary material for: Hearing problems in humans and mouse models with rare copy number variants associated with schizophrenia: a scoping review protocol
Source: Wellcome Open Res. 2024 Dec 16;9:546. Originally published 2024 Sep 24. [Version 2] doi: 10.12688/wellcomeopenres.23013.2 (PMC11809143; doi:10.12688/wellcomeopenres.23013.2)
Supplement: Peer review history

# Reviewer report: Reviewer response for version 2

**DOI:** 10.21956/wellcomeopenres.25944.r115734

**Published:** 2024-12-17

**Authors:** Esra Sefik

**Affiliations:** 1 Princeton Neuroscience Institute, Princeton University, Princeton, New Jersey, USA

The authors have adequately addressed my previous comments. I have no additional feedback at this time and wish them the best of luck with their study.

Is the study design appropriate for the research question?

Partly

Is the rationale for, and objectives of, the study clearly described?

Yes

Are sufficient details of the methods provided to allow replication by others?

Partly

Are the datasets clearly presented in a useable and accessible format?

Not applicable

Reviewer Expertise:

My background includes research on schizophrenia-associated copy number variants, specifically the 3q29 and 22q11.2 deletions, and their connection to psychosis risk. I have experience working with both human and mouse model data in this area, as well as involvement in non-genetic, clinical high-risk states for psychosis, where I have focused on early indicators and risk factors for the onset of psychotic disorders among at-risk youth. I also have methodological expertise in conducting meta-analyses and systematic reviews, which I have applied to synthesizing research findings and establishing normative benchmarks for typical brain development, as well as for neurodevelopmental and psychiatric conditions such as schizophrenia and autism.

I confirm that I have read this submission and believe that I have an appropriate level of expertise to confirm that it is of an acceptable scientific standard.

# Reviewer report: Reviewer response for version 2

**DOI:** 10.21956/wellcomeopenres.25944.r115733

**Published:** 2024-12-17

**Authors:** Clara S Humpston

**Affiliations:** 1 University of York, Heslington, UK

Many thanks for addressing my previous comments. I have no more feedback and am very happy to approve.

Is the study design appropriate for the research question?

Yes

Is the rationale for, and objectives of, the study clearly described?

Yes

Are sufficient details of the methods provided to allow replication by others?

Yes

Are the datasets clearly presented in a useable and accessible format?

Not applicable

Reviewer Expertise:

Schizophrenia-spectrum psychosis, auditory and auditory-verbal hallucinations, psychopharmacology, phenomenology of psychosis

I confirm that I have read this submission and believe that I have an appropriate level of expertise to confirm that it is of an acceptable scientific standard.

# Reviewer report: Reviewer response for version 1

**DOI:** 10.21956/wellcomeopenres.25343.r109259

**Published:** 2024-11-07

**Authors:** Clara S Humpston

**Affiliations:** 1 University of York, Heslington, UK

This is a well-written protocol for a scoping review, which aims to explore whether certain CNVs associated with schizophrenia are related to various forms of hearing problems in both humans and mouse models.

 I am confident that this protocol is methodologically sound, although conceptually I would like to see some clarifications or further information on the following points:

 1) Auditory hallucinations are not always psychosis symptoms - I think it needs to be made clearer that auditory hallucinations are by no means pathognomonic of schizophrenia or any psychotic disorder, and there are many differences between auditory hallucinations broadly (including hearing noise, music, etc.) and auditory-verbal hallucinations (hearing speech). 

 2) To what extent do the CNVs confer genetic risks for schizophrenia, as opposed to hearing hallucinated sounds or voices more generally? This is related to my previous point about the specificity of hallucinations as a symptom of schizophrenia.

 3) Delirium is not usually considered a psychotic symptom, and it may confuse or dilute the focus of this scoping review if too many related symptoms are mixed into the conceptualisation or perhaps even methods.

 4) Research question 1) asks whether CNVs that increase the risk of schizophrenia are associated with hearing problems, but insufficient information is mentioned about whether these CNVs also increase the risk of auditory hallucinations more specifically. 

 I hope the authors find these comments helpful.

Is the study design appropriate for the research question?

Yes

Is the rationale for, and objectives of, the study clearly described?

Yes

Are sufficient details of the methods provided to allow replication by others?

Yes

Are the datasets clearly presented in a useable and accessible format?

Not applicable

Reviewer Expertise:

Schizophrenia-spectrum psychosis, auditory and auditory-verbal hallucinations, psychopharmacology, phenomenology of psychosis

I confirm that I have read this submission and believe that I have an appropriate level of expertise to confirm that it is of an acceptable scientific standard, however I have significant reservations, as outlined above.

# Response

**Published:** 2024-12-09

**Authors:** Stephen Murtough

**Affiliations:** Division of Psychiatry, University College London, London, UK

Thank you, Clara, for your kind and helpful comments. We provide our responses below.

 1) We agree that we should provide clarification that some auditory hallucinations are not associated with psychosis or schizophrenia, and we have added the following sentence to the main text: “The mechanisms that drive these processes remain largely unexplored, and an opportunity could be to study individuals who are at high genetic risk of schizophrenia; although we note that auditory hallucinations can occur in people who do not have schizophrenia, and they can vary from hearing a simple sound to complex speech.”

 2) We thank you for your comments on whether the 13 CNVs specifically increase a person’s risk of auditory hallucinations. The 13 CNVs were identified using a genome-wide approach comparing schizophrenia cases and controls (Marshall *et al*, 2017). No data or estimates were provided in the publication (to the best of our knowledge) about risk of experiencing auditory hallucinations. Therefore, we cannot determine the precise risk that these CNVs may carry for experiencing auditory hallucinations. We have noted this as a limitation in the conclusion of the main text: “A further limitation is that while the 13 CNVs are associated with schizophrenia, it is not known whether they specifically increase a person’s risk of auditory hallucinations, which is the symptom most observed in individuals with hearing loss. We assume that individuals with a high genetic risk of schizophrenia are more likely to experience auditory hallucinations; however, we are aware that auditory hallucinations can vary from hearing a simple sound to complex speech, and they are not necessarily associated with psychosis and schizophrenia.”

 3) We agree that delirium should not be listed as a psychosis symptom, and we have removed all mentions of this in the main text.

# Reviewer report: Reviewer response for version 1

**DOI:** 10.21956/wellcomeopenres.25343.r102571

**Published:** 2024-11-04

**Authors:** Biju Viswanath

**Affiliations:** 1 National Institute of Mental Health and Neurosciences, Bengaluru, India

The list of CNVs is based on a 2017 paper.

 Are there additional CNVs in the last 7 years? Are there any identified CNVs based on non-European samples? These are important considerations for this scoping review to be useful

 There is some confusion regarding English vs Non-English manuscripts. There is some reference to translation using DeepL

 A flowchart describing the overall plan would be useful

Is the study design appropriate for the research question?

Yes

Is the rationale for, and objectives of, the study clearly described?

Yes

Are sufficient details of the methods provided to allow replication by others?

Yes

Are the datasets clearly presented in a useable and accessible format?

Not applicable

Reviewer Expertise:

Psychiatry, Genetics, Stem cell biology

I confirm that I have read this submission and believe that I have an appropriate level of expertise to confirm that it is of an acceptable scientific standard, however I have significant reservations, as outlined above.

# Response

**Published:** 2024-12-09

**Authors:** Stephen Murtough

**Affiliations:** Division of Psychiatry, University College London, London, UK

Thank you, Biju, for your helpful comments. We provide our responses below.

1. The 2017 publication from Marshall *et al* is a landmark study from the CNV and Schizophrenia Working Groups within the Psychiatric Genomics Consortium.  This is the most recent and comprehensive genome-wide analysis of CNVs that increase risk of schizophrenia, comprising 21,094 cases and 20,227 controls. We consider this to be the most robust publication to base our scoping review on, although we agree that there is substantial bias for European samples. To mark this as a limitation, we have included the following sentence in the main text:
  “We note, however, that these CNVs were identified using European samples, and other schizophrenia-associated CNVs in individuals with different ancestries are likely to be under-reported.”
2. We appreciate your comments regarding the language of articles to be included in the review. To clarify, no language restrictions will be applied during the literature search, although searches will be performed using English. Any articles that are not written in English will be translated into English using DeepL, which is an online translating software. To provide clarity for the reader, we have added an extra sentence to the main text, confirming that the literature search will be performed using English.
3. We thank you for your suggestion to include a flow chart as a visual aid. In this instance, we have decided not to include this, as we consider the protocol to be clear and concise in its current format. However, we intend to produce a detailed flow chart for the scoping review publication, which will include information about the number of studies identified during the selection process.  

# Reviewer report: Reviewer response for version 1

**DOI:** 10.21956/wellcomeopenres.25343.r103673

**Published:** 2024-10-25

**Authors:** Esra Sefik

**Affiliations:** 1 Princeton Neuroscience Institute, Princeton University, Princeton, New Jersey, USA

This scoping review protocol focuses on an intriguing but understudied intersection between hearing impairment, its epidemiological association with psychosis symptoms (including auditory hallucinations), and genetic risk for psychosis, specifically through the study of 13 CNV loci that are known to confer increased risk for schizophrenia, eight of which have reached genome-wide significance in the landmark study by Marshall et al. (2017). While 22q11.2 deletion is the most extensively studied loci among these, with previously documented hearing impairments in both humans and mouse models, hearing problems in other schizophrenia-associated CNVs remain largely unexamined, and no comprehensive review of the literature exists on this subject. The study protocol outlines two primary research questions to address this gap: 1) Are CNVs that increase risk of schizophrenia associated with hearing problems in human and mouse studies?, and 2) If hearing problems are observed, what is their nature?. The authors posit that if hearing problems are a clinical feature in these groups (including humans and related mouse models), they may serve as useful genetic models for future mechanistic studies.

 This is a compelling research area that merits further exploration, and the rationale is clearly articulated. The inclusion of studies on both human and mouse models offers cross-species insights and has translational value. The methodology for literature review is well-designed and adheres to established guidelines (such as Joanna Briggs Institute and PRISMA-ScR), ensuring a structured and systematic approach to synthesizing existing evidence on this topic. The use of a clear search strategy across four databases (PubMed, Embase, PsychINFO, Cochrane Library), independent screening by multiple reviewers, the inclusion of a forward citation search approach, and consideration of multiple forms of hearing impairment while distinguishing between central and peripheral forms of hearing impairment, promotes rigor.

**I cannot wait to see the results; however, I have some comments:**

 The abstract states, “we hypothesise that hearing impairment is an understudied mechanism that contributes to the formation of psychosis symptoms in individuals at high genetic risk.”  In its current form, the study is well-suited to identify associations between schizophrenia-associated CNVs and hearing issues, but it is not designed to test whether hearing impairments themselves contribute to the onset of psychosis and/or exacerbate the severity of psychotic symptoms in these CNVs. This is why I would caution against the use of the phrase "contributes to the formation of psychosis" in your formal hypothesis. It is important to recognize that these 13 CNVs are linked to a wide range of clinical manifestations and show high comorbidity, contributing to an increased risk for multiple neurodevelopmental and psychiatric disorders such as ASD (also exhibits higher rates of hearing impairment than the general population; see Rosenhall et al., 1999; Tas et al., 2007), ADHD, intellectual disability etc. Not all individuals carrying these CNVs develop psychosis, and an increased prevalence of hearing impairments in these genetically at-risk populations does not necessarily indicate a direct contribution of hearing problems to the development of psychosis as a clinical outcome. Adjusting the framing of the hypothesis to reflect this limitation would better align with the study's scope and design. Alternatively, the human arm could be restructured to formally test for correlations between hearing impairment (presence/absence or severity) and psychotic symptoms (using either categorical or dimensional approaches) among individuals with schizophrenia-associated CNVs to probe this hypothesis more directly. However, this would necessitate not only a more detailed data extraction protocol but also the availability of studies where patient-level data on both hearing impairment and psychiatric screening are available. Without access to such granular data, drawing meaningful correlations between these variables may not be feasible.

 Odds ratios for the 13 schizophrenia-associated CNVs included in the study are extracted from Marshall et al. (2017) and reported in Table 1. I recommend adding the 95% confidence intervals for these odds ratios as reported in the original study to provide an estimate of the uncertainty around these numbers. Additionally, I suggest changing the "not available" notation for the 3q29 deletion to "infinite" in this table, as this is more consistent with Marshall et al. (2017)'s original report. The odds ratio that was labeled as "infinite" in the referenced publication is indeed based on available data (16 schizophrenia cases vs. 0 controls) and reflects an exceptionally high risk for schizophrenia associated with the 3q29 deletion; therefore, "not available" is not an accurate representation of the corresponding result for this CNV. You could also consider complementing the information provided for this CNV in Table 1 with additional estimates from other relevant studies, such as those reported by Mulle, 2015. This could be done for other CNVs too, to capture the variability or consistency of risk estimates across different studies.

 A sample PubMed search string for 22q11.2 is provided in Table 2. To ensure the reproducibility of search results, similar search strings should be provided for all included CNVs, either in the extended data or within the main manuscript.

 The data analysis section could benefit from further elaboration to clarify the meta-analysis approach that will be employed if sufficient studies with comparable quantitative outcomes are identified. It would be helpful to outline how the study plans to address key considerations such as publication bias, sensitivity analyses, potential sub-group analyses, fixed-effect vs random-effects models for meta-analysis etc. Clarifying these aspects will enhance the methodological transparency and rigor of the meta-analysis, should it be feasible to move beyond a summary-based narrative approach.

 I wish the research team the very best for this project and look forward to seeing the results of this work.

Is the study design appropriate for the research question?

Partly

Is the rationale for, and objectives of, the study clearly described?

Yes

Are sufficient details of the methods provided to allow replication by others?

Partly

Are the datasets clearly presented in a useable and accessible format?

Not applicable

Reviewer Expertise:

My background includes research on schizophrenia-associated copy number variants, specifically the 3q29 and 22q11.2 deletions, and their connection to psychosis risk. I have experience working with both human and mouse model data in this area, as well as involvement in non-genetic, clinical high-risk states for psychosis, where I have focused on early indicators and risk factors for the onset of psychotic disorders among at-risk youth. I also have methodological expertise in conducting meta-analyses and systematic reviews, which I have applied to synthesizing research findings and establishing normative benchmarks for typical brain development, as well as for neurodevelopmental and psychiatric conditions such as schizophrenia and autism.

I confirm that I have read this submission and believe that I have an appropriate level of expertise to confirm that it is of an acceptable scientific standard, however I have significant reservations, as outlined above.

## References

[1] : Autism and hearing loss. *J Autism Dev Disord* .1999;29(5) : 10.1023/a:1023022709710 349-57 10.1023/a:1023022709710 10587881

[2] : Evaluation of hearing in children with autism by using TEOAE and ABR. *Autism* .2007;11(1) : 10.1177/1362361307070908 73-9 10.1177/1362361307070908 17175575

[3] : The 3q29 deletion confers >40-fold increase in risk for schizophrenia. *Molecular Psychiatry* .2015;20(9) : 10.1038/mp.2015.76 1028-1029 10.1038/mp.2015.76 PMC454652926055425

# Response

**Published:** 2024-12-09

**Authors:** Stephen Murtough

**Affiliations:** Division of Psychiatry, University College London, London, UK

Thank you, Esra, for your kind and thoughtful review. We appreciate all your points for improvement, and our responses are below.

1. We agree that our study is not well designed to test our previously specified hypothesis. We have amended the abstract text to include the following: “We hypothesise that individuals who have a high genetic risk of schizophrenia may also experience hearing problems”. We have also amended the main text to reflect the new hypothesis.
2. We appreciate your suggestion to formally test for correlations between hearing problems and psychosis symptoms. Our assumption, based on a preliminarily literature search, is that data will not be available in sufficient detail to allow for this approach, and therefore we have decided not to include this in our protocol. If we find that sufficient data are available, however, we will update the study protocol to include this information.
3. We agree that 95% confidence intervals should be added to Table 1, and that the odds ratio for the 3q29 deletion should be amended to “infinite”, as described in the original publication by Marshall *et al*. We have made these changes to Table 1 in the manuscript.
4. We agree that search strategies should be provided for all 13 schizophrenia-associated CNVs. We have provided these as Appendix 2 in the extended data, and we have referred to this in the main text.
5. We welcome your suggestion to provide more detail about the meta-analysis strategy. Based on a preliminary literature search, our assumption is that insufficient data will be available to complete a meta-analysis. Therefore, we are inclined to keep our protocol focused as a scoping review, and we have removed all mentions in the main text about performing a meta-analysis. If we find that sufficient data are available after the full literature search, we will update the study protocol with this information.
